# Incidence, mortality, and the importance of drug therapy in cannabis-induced psychosis

**DOI:** 10.1192/j.eurpsy.2025.46

**Published:** 2025-08-26

**Authors:** H. Taipale

**Affiliations:** Department of Clinical Neuroscience, Karolisnka Institutet, Stockholm, Sweden

## Abstract

**Abstract:**

Introduction

Cannabis-induced psychosis (CIP) is often assumed to resolve with sustained abstinence, although recent studies report adverse long-term outcomes, such as a high risk of diagnostic transition to schizophrenia. Cannabis is also known to increase relapse rate in individuals with psychotic disorders in general, and there is a lack of knowledge on optimal treatment of this patient group.

**Objectives:**

In this presentation, data on incidence and mortality associated with CIP, and real-world effectiveness of antipsychotics in relapse prevention in persons with CIP will be presented.

**Methods:**

Nationwide Scandinavian registers were utilized to identify persons with first-time CIP (ICD-10 F12.5) during the years 2000-2021. Incidence and mortality of CIP were investigated with Danish, Norwegian and Swedish register-based data, and antipsychotic use with Swedish data. Annual incidence rates of CIP per 100 000 persons were calculated. For mortality study, incident cases of CIP were matched by age- and gender with comparison persons without substance-induced psychosis and hazard ratios (HRs) were calculated for all-cause and cause-specific mortality. Association between use of specific antipsychotics (oral vs. long-acting injectable, LAI, forms) and risk of hospitalization due to any psychosis relapse was investigated in within-individual design where each person acted as his/her own control to minimize selection bias, analyzed with stratified Cox models.

**Results:**

The incidence rate of CIP increased in time in all Scandinavian countries and was the highest in Denmark throughout the study years (rate in 2016 5.6, vs. 3.0 in Norway and 2.7 in Sweden). CIP was associated with an increased risk of mortality, risk estimates (HRs) ranging from 6.6 in Denmark, to 7.6 in Sweden, and 9.0 in Norway, compared with general population controls. In within-individual models, antipsychotic use was associated with a decreased risk of hospitalization due to psychosis relapse, with an adjusted HR of 0.75 (95% Confidence Interval 0.67-0.84), compared with time periods when the same individuals did not use antipsychotics.

**Conclusions:**

Incidence of cannabis-induced psychosis is increasing in Scandinavian countries, and it is associated with significant mortality risk. Antipsychotics are effective treatments in preventing psychosis relapses also in individuals with CIP.

**Disclosure of Interest:**

H. Taipale Grant / Research support from: Janssen, Speakers bureau of: Gedeon Richter, Janssen, Lundbeck, Otsuka

